# Nanoindentation and Structural Analysis of Sintered TiAl*_(100−x)_*-*_x_*TaN Composites at Room Temperature

**DOI:** 10.3390/ma16072607

**Published:** 2023-03-24

**Authors:** Bukola Joseph Babalola, Olusoji Oluremi Ayodele, Peter Apata Olubambi

**Affiliations:** Centre for Nanoengineering and Advanced Materials, School of Mining, Metallurgy and Chemical Engineering, University of Johannesburg, Johannesburg 2028, South Africa

**Keywords:** titanium aluminide (TiAl), tantalum nitride (TaN), spark plasma sintering, composites, nanoindentation

## Abstract

The nanohardness, elastic modulus, anti-wear, and deformability characteristics of TiAl*_(100−x)_*-*_x_*TaN composites containing 0, 2, 4, 6, 8, and 10 wt.% of TaN were investigated via nanoindentation technique in the present study. The TiAl*_(100−x)_*-*_x_*TaN composites were successfully fabricated via the spark plasma sintering technique (SPS). The microstructure and phase formation of the TiAl sample constitute a duplex structure of γ and lamellar colonies, and TiAl_2_, α-Ti, and TiAl phases, respectively. The addition of TaN results in a complex phase formation and pseudo duplex structure. The depth-sensing indentation evaluation of properties was carried out at an ambient temperature through a Berkovich indenter at a prescribed load of 100 mN and a holding time of 10 s. The nanoindentation result showed that the nanohardness and elastic modulus characteristics increased as the TaN addition increased but exhibited a slight drop when the reinforcement was beyond 8 wt.%. At increasing TaN addition, the yield strain (HEr), yield pressure (H3Er2), and elastic recovery index (WeWt) increased, while the plasticity index (WpWt) and the ratio of plastic and elastic work (RPE) reduced. The best mechanical properties were attained at the 8 wt.%TaN addition.

## 1. Introduction

Titanium aluminides (TiAl) have drawn much attention due to their high specific strength, good creep resistance, low density [1], oxidation resistance within 800–900 °C, and high melting point [2], which makes them the ideal candidate material for lightweight structures in the aerospace and automotive industry [3]. After decades of extensive research and development, advanced, typical TiAl alloys such as the Ti-48Al-2Cr-2Nb alloys have been utilized for Boeing 747-8 aircraft, specifically in the sixth and seventh turbine blades of GEnx^TM^-2B aero-engines. Likewise, the application of Ti-43.5Al-4Nb-1Mo-0.1B, also known as TNM alloy, has been used for application in the low-pressure turbine blade in the PW1100G aero-engines of Airbus A320neo aircraft, with an application temperature range of 600–800 °C [3,4]. In spite of the merits mentioned above, TiAl alloys exhibit poor plasticity at room temperature (<2%) [4,5,6] and a reduction in strength at elevated temperatures [3].

Strategies to circumvent these problems have been explored through several kinds of research studies, ranging from alloying [7], composite development [8,9], processing technology [10,11], and heat treatment processes [4]. Among these strategies, the development of composite materials has attracted enormous interest due to the complementary benefits of matrix and reinforcing materials making up the composite. There are various reinforcing materials used for the development of composites proven to enhance properties [12], such as B_4_C [13], TiC [14], Ti_2_AlC [14,15], SiC [16], TiB_2_ [17], oxides [18], and nitrides [19]. The use of these reinforcing materials in enhancing the chemical, physical, and mechanical properties has been documented in research work. Tengfei et al. [20] investigated the microstructure and mechanical properties of spark plasma sintered micro-nano Ti_2_AlC reinforced TiAl composites. The authors reported that the incorporation of the micro-nano Ti_2_AlC enhanced the tribological properties of the composites at room temperature. Significant improvement was also observed in the compressive strength and plasticity of the composites. Kan et al. [21], on the development of nano-TiC reinforced Nb-TiAl composites via electron beam melting, reported that the microhardness of the developed composite was 24% higher than the matrix. In work undertaken by Yingmei et al. [22], a significant improvement in the compressive strength and strain of the composite was observed at 0.9% SiC addition, with enhanced strength and strain at 1383 MPa and 6.5%, respectively. Wei et al. [23] observed a gradual decrease in the average grain size and transformations in the crystallographic orientation of TiAl/TiB_2_ in situ in a metal matrix composite developed via selective laser melting (SLM). In addition, significant nanohardness was observed at 10.57 ± 0.53 GPa, a higher value compared to conventionally produced TiB_2_ reinforced TiAl alloy. With respect to microstructural refinement, an alloying element such as Ta is very effective, which consequently enhances tensile strength and plasticity when added to a TiAl alloy [24,25,26]. Meanwhile, reinforcing with nitride particles has been proven to impact superior mechanical properties and to be an efficient reinforcing material for TiAl alloys [19]. Tantalum nitride (TaN) is a typical nitride material which exhibits good mechanical properties with the highest microhardness attributes among transition metal nitrides and affords them excellent wear resistance capabilities [27]. It also possesses good chemical inertness and high-temperature stability. However, there have been limited or no reports on the addition of TaN to TiAl matrix alloys.

In addition to improving the TiAl matrix alloy via composite technology, the processing route also influences the microstructure, which in turn affects the properties. Therefore, attaining the material’s intrinsic structure and properties is a function of the processing route. The production of TiAl alloys and composites possesses very daunting challenges. For instance, melts reacting with the crucible in a vacuum induction melting [28] result in the end product’s chemistry, structural, and property attributes being compromised. In addition to the above, the high melting temperature of intermetallics makes it difficult to have rapid solidification techniques, coupled with high reactivity of melts and bad densification [28]. A novel fabrication method for TiAl alloys and composites is the Spark Plasma Sintering (SPS) technique. SPS offers the ability to fabricate alloys and composites with high densification of constituent powders within a relatively short processing time [29,30] and lower temperature, and also prevents impurities from reacting with the component [31,32,33,34].

Consequently, the nanoindentation technique offers distinct advantages compared to conventional indentation methods [35]. This technique has been used by researchers [31,36,37,38,39,40,41,42,43,44] to shed light on various mechanical property responses of materials. It offers the unique advantage of evaluating materials’ properties without compromising the structure of a material.

In this study, the microstructure and nanoindentation responses of the sintered TiAl alloy and composites developed via spark plasma sintering technique were studied using scanning electron microscopy (SEM) and nanoindentation techniques, respectively.

## 2. Materials and Methods

### 2.1. Materials and Fabrication

Ti-48Al-2Cr-2Nb (TiAl) (at. %) and TaN powders were selected as the matrix and reinforcement materials, respectively, to develop the (TiAl*_(100−x)_*-*_x_*TaN) composites used in this study. The details of the powder materials are presented in Table 1. The addition of TaN was varied at 0, 2, 4, 6, 8, and 10 wt.%, weighed, and mixed with the corresponding content of TiAl as a balance to attain homogeneity in a tubular mixer for 8 h. The mixed powers were consolidated via the SPS technique (HHPD-25 Model, FCT Systeme, GmbH, Germany) at constant sintering parameters, constituting a temperature of 1150 °C, pressure of 50 MPa, 10 min dwell time and a heating rate of 100 °C/min. The temperature within the graphite punch was monitored with an external pyrometer. Sintered components of cylindrical shape with a dimension of 5 mm height and 30 mm diameter were obtained after the sintering process.

### 2.2. Microstructural and Phase Evaluation

After the sintered composites were removed from the sintering machine, the graphite foil on their surfaces was removed via sandblasting. This was followed by grinding, polishing, and etching using Kroll’s reagent for metallography. The phase and structural investigations were conducted on the sintered composites using an X-ray diffractometer (PW1710 Philips, PANalytical Empyrean XRD model, Malvern, UK) with Cu Kα radiation (X-ray wavelength, λ = 0.15406 nm) and scanning electron microscopy (FESEM JEOL JSM-7900F, Welwyn Garden City, UK) equipped with energy dispersive X-ray spectroscopy (EDX) for elemental analysis, respectively. The Williamson–Hall method was utilized to compute the lattice strain, crystallite size, and dislocation density of the sintered samples. As suggested by this method, the total broadening, $\beta_{hkl}$, comprises the broadening from lattice strain, $\beta_{\varepsilon }$, and crystallite size, $\beta_{D}$, detected in the X-ray [45]. The Williamson–Hall method can be computed as follows:

(1) βhkl=βD+βε=KλDcosθ+4εtanθ

In order to execute the Williamson–Hall plot, Equation (1) is simplified as follows:

(2) βhklcosθ=4εsinθ+KλD

Here, the $\beta_{hkl}$ represents the diffraction peak width at half maximum intensity in radians (FWHM), *K* represents the shape factor (0.9), *D* is the crystallite size, *ɛ* is the lattice strain, *ϴ* means the Bragg’s angle, which is the peak position in radians, and *λ* is the wavelength (0.15406 nm).

### 2.3. Nanoindentation of Sintered Samples

The as-sintered samples with 30 mm in diameter and 5 mm in height were mirror-finished for the nanoindentation test. The nanoindentation properties of the sintered composites were determined by an Anton Paar Hit 300 nanoindentation tester with a Berkovich diamond indenter in compliance with ASTM E2546 [46]. The loading rate, load-hold, and unloading rate were constant for all sintered samples. During the test, a maximum applied load of 100 mN was applied with a dwell time of 10 s set between the loading and the unloading state of the test. The system recorded the indentation load and penetration depth with respect to time. An average of 10 indentations for each of the sintered samples was recorded in this study. The load and displacement data from the system were evaluated using the Oliver and Pharr principle [47]. According to Oliver and Pharr [47], the reduced elastic modulus (*E_r_*) and hardness (*H*) can be obtained via the following equations.

(3) H=PmaxAc

where *P_max_* represents the maximum load and the indentation area is *A_c_*. The indentation area, *A_c_*, is expressed as

(4) $$A_{c}=P(h_{c}^{2})$$

where the applied load is represented by *P* and *h_c_* represents the contact depth at the maximum load. The relationship between the contact depth (*h_c_*), maximum depth (*h_max_*), peak load (*P_max_*), the geometry constant of the indenter (*ɛ*), and the contact stiffness (*S*) can be calculated by the equation below:

(5) hc=hmax−εPmaxS

The contact stiffness (*S*) can be calculated by the equation stated below:

(6) S=dPdh=β2πErAc

where *P* and *h* represent the applied load and depth of penetration during indentation, respectively. The constant related to the geometry of the indenter, which is represented by *β*, is expressed as 1.034 for a Berkovich indenter [48,49], and *E_r_* is the reduced elastic modulus defined as:

(7) $$\frac{1}{E_{r}}=\frac{1-v^{2}}{E}+\frac{1-v_{i}^{2}}{E_{i}}$$

Here, (*E*) and (*v*) are Young’s modulus and Poisson’s ratio of the sample, and *E_i_* and *v_i_* represent that of the Berkovich indenter, respectively [44]. The Berkovich indenter has an elastic constant and Poisson’s ratio of 1141 GPa and 0.07, respectively [48]. In addition, the total work carried out by the indenter (*W_t_*) gives insight into the mechanical attributes of the sintered composites by evaluating their elastic index [WeWt] and plastic index [WpWt]. The total work carried out by the indenter can be calculated by the area under the load–displacement nanoindentation curve as:

(8) $$W_{t}=W_{e}+W_{p}$$

where $W_{e}$ represents the elastic energy and $W_{p}$ represents the plastic energy. 

## 3. Results and Discussion

### 3.1. Microstructural and Phase Analysis

**Figure 1:**
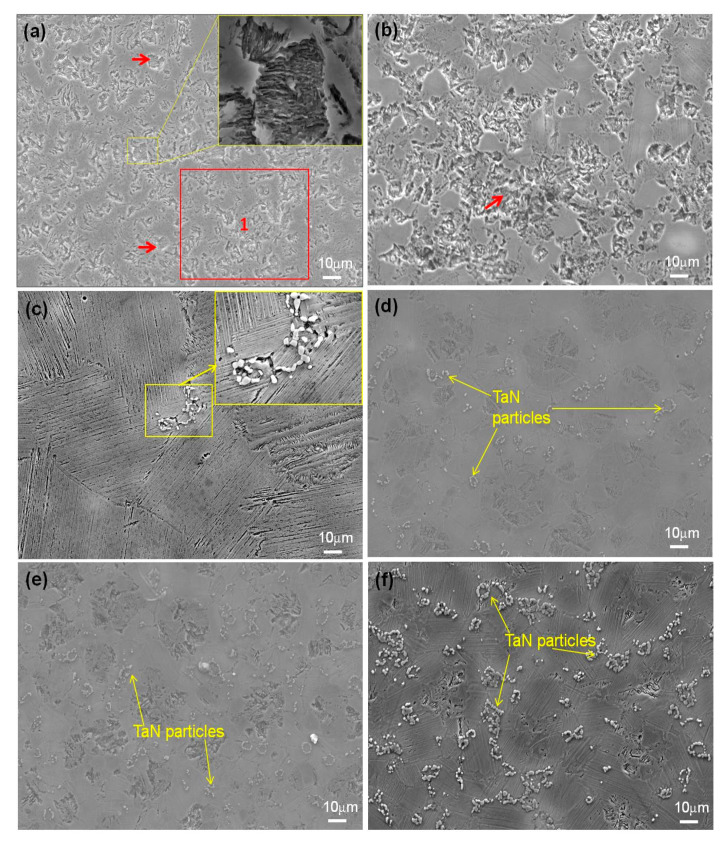
SEM images of sintered samples: (**a**) pure TiAl, (**b**) TiAl−2 wt.%TaN, (**c**) TiAl−4 wt.%TaN, (**d**) TiAl−6 wt.%TaN, (**e**) TiAl−8 wt.%TaN, and (**f**) TiAl−10 wt.%TaN.

**Figure 2:**
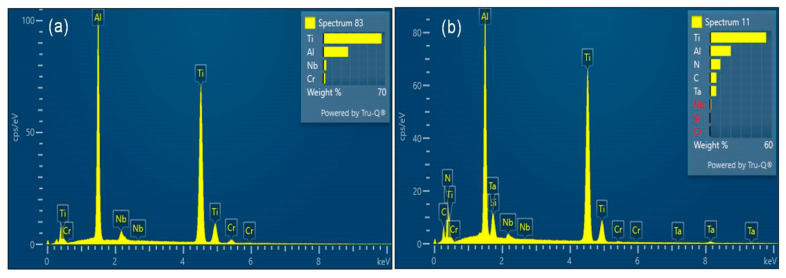
ESD spectrum analysis: (**a**) rectangular region denoted by 1 in Figure 1a, and (**b**) TiAl−2 wt.% TaN.

The SEM images of the sintered samples consisting of the pure TiAl and TiAl*_(100−x)_*-*_x_*TaN composites are presented in Figure 1. As shown in the SEM images of the sintered samples in Figure 1a, the sintered TiAl powder depicts the distribution of a typical duplex structure constituting γ phase and lamellar colonies, indicated by red arrows (with higher magnification shown as insets, Figure 1a). This distinct microstructure has been reported to exhibit the mechanical properties of both phase constituents, with more ductile structure and therefore preferred for structural applications [50]. The EDS analysis confirming the chemical composition is presented in Figure 2a.

Figure 1b shows the SEM image of reinforced TiAl with 2 wt.% of TaN, which reflects a similar structure to the pure TiAl microstructure. The EDS analysis is presented in Figure 2b, indicating no inclusions in the composite. At 4 wt.% TaN (Figure 1c), the microstructure depicts a lamellar structure with obvious TaN particles concentrated at the grain boundary. The inset of Figure 1c reveals the morphology of the TaN particles at the grain boundary. Owing to the fact that the lamellar structure exhibits poor ductility at room temperature, the provision of small particles with the structure enhances its mechanical and ductility characteristics [50]. However, at higher concentrations of TaN, the microstructure exhibited more of a typical pseudo duplex structure and even distribution of the TaN particles. This implies that the TaN addition could facilitate the transformation of the duplex structure to a pseudo-duplex structure. The pseudo-duplex structure constitutes more of the γ phase [50,51]. Generally, it can be seen that the microstructure exhibits an even distribution of clustered TaN particles at the grain boundaries with increasing addition.

**Figure 3:**
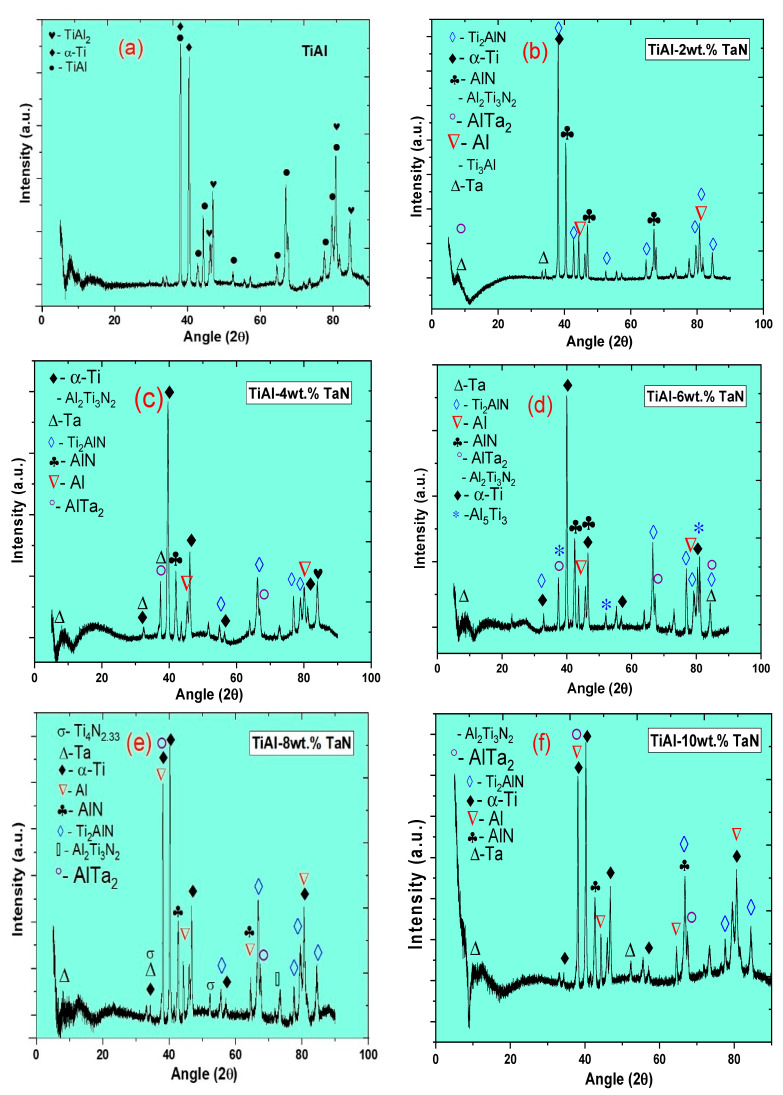
X-ray diffraction patterns of sintered samples at a sintering temperature of 1050 °C, pressure of 50 MPa, dwell time of 10 min, and heating rate of 100 °C/min: (**a**) pure TiAl sample, (**b**) TiAl−2wt.% TaN, (**c**) TiAl−4wt.% TaN, (**d**) TiAl−6wt.% TaN, (**e**) TiAl−8wt.% TaN, and (**f**) TiAl−10wt.% TaN.

**Figure 4:**
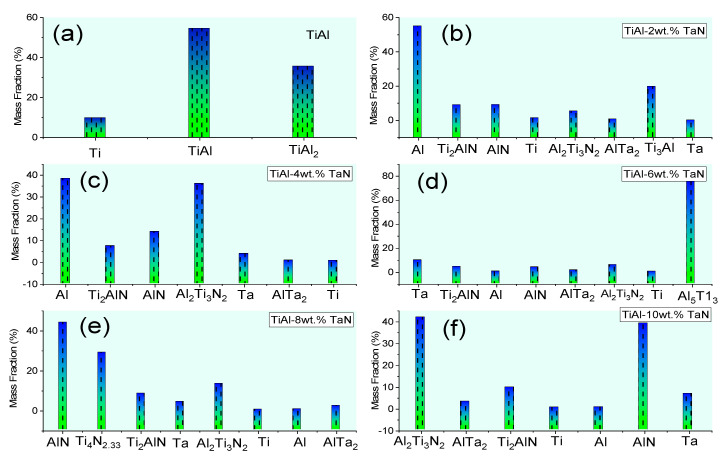
Variation of the mass fraction of phases in the sintered samples: (**a**) TiAl, (**b**) TiAl−2wt.%TaN, (**c**) TiAl−4wt.%TaN, (**d**) TiAl−6wt.%TaN, (**e**) TiAl−8wt.%TaN, and (**f**) TiAl−10wt.%TaN.

Figure 3 shows the XRD patterns of the sintered samples. Figure 3a shows the XRD result of pure TiAl, which indicates that the sample has three phases of TiAl_2_ (Cmmm), α-Ti (P63/mmc), and γ-TiAl (P4/mmm). With the addition of TaN, more phases were precipitated. Conversely, γ-TiAl and TiAl_2_, as present in the pure TiAl sample, were not detected in the sintered composites. These may have evolved into Ti_3_Al (P63/mmc), Al_5_Ti_3_ (Pmmm), Ti_2_AlN (P63/mmc), hexagonal Ti_4_N_2.33_ (R-3m), and Al_2_Ti_3_N_2_ (P31c) phases, as shown in Figure 3b–f. Other phases detected were AlTa_2_ (P42/mnm), AlN (Fm-3m), and Ta, Ti, and Al elements. These phases were detected in all the sintered composites except for Ti_3_Al, Al_5_Ti_3_, and Ti_4_N_2.33_, which were only detected in samples with 2, 6, and 8 wt.% TaN additions, respectively. A detailed observation shows that the phases formed in all the sintered samples do not contain any oxides, which confirms that there is no contamination associated with the sintering of the samples. The crystallite size of the sintered samples, as deduced from the XRD patterns (Table 2), reduces with increasing TaN addition. In contrast, the lattice strain and dislocation density increase, indicating a refined microstructure. In order to quantify the mass fraction of phases obtained in the sintered materials, Reference Intensity Ratio (RIR) analysis was utilized and summarized in Figure 4. It can be seen that the γ-TiAl exhibited the highest mass fraction of 54.6% in the pure TiAl sample. At 2 wt.% TaN, Al, Ti_3_Al, and AlN obtained 55.03%, 19.68%, and 9.07% mass fractions, respectively. Al, Al_2_Ti_3_N_2_, and AlN phases obtained 38.25%, 35.85%, and 13.82% mass fractions at 4 wt.% TaN addition, respectively. At 6, 8, and 10 wt.% TaN addition, Al_5_Ti_3_, AlN, and Al_2_Ti_3_N_2_ obtained 75.73%, 43.72%, and 41.64% mass fractions, respectively. While the mass fractions of the phases according to increasing TaN addition did not follow any regular trend, the complexity of phase formation within the composites can be attributed to TaN addition.

### 3.2. Nanoindentation Analysis of the Mechanical Properties of the Sintered Samples 

#### 3.2.1. Nanoindentation Load–Depth and Depth–Time Curves of the Sintered Samples

**Figure 5:**
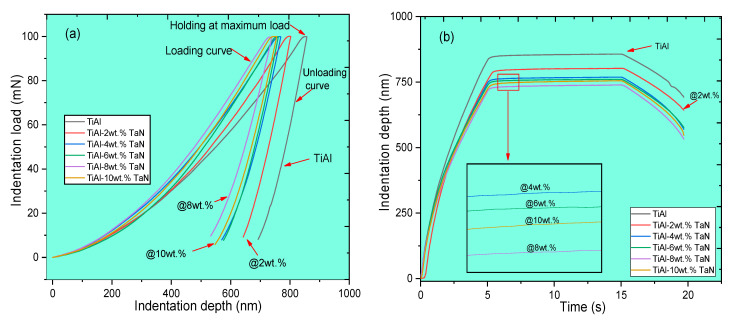
Characteristic load–depth curves (**a**) and depth–time curves (**b**) for the sintered TiAl, and TiAl-based composites.

The nanoindentation load–depth and depth–time curves for the sintered pure TiAl and TiAl*_(100−x)_*-*_x_*TaN (x = 2, 4, 6, 8, and 10) composites at 100 mN load are presented in Figure 5. The load–depth curves for the sintered samples are presented in Figure 5a, consisting of three major segments: loading, holding at maximum load, and unloading. The curves exhibited by the samples are smooth, with no appearance of the pop-in effects. The sintered TiAl sample reveals the highest indentation depth, while the indentation depth reduces as the TaN addition increases. The lowest indentation depth of 732.21 nm was attained at 8 wt.%TaN addition and increased to 745.64 nm at 10 wt.% TaN addition, and the TiAl sample recording the highest depth of 854.53 nm, as presented in Figure 5b. This shows that increasing the addition of TaN promotes an increasing resistance to plastic deformation during indentation when compared to the sintered TiAl sample. Consequently, the reductions in the indentation depth indicate that the sintered composite samples exhibit enhanced hardness and stiffness due to load transfer from the matrix to reinforcement.

#### 3.2.2. Nano-Hardness and Elastic Modulus of the Sintered TiAl and TiAl*_(100−x)_*-*_x_*TaN Composites

**Figure 6:**
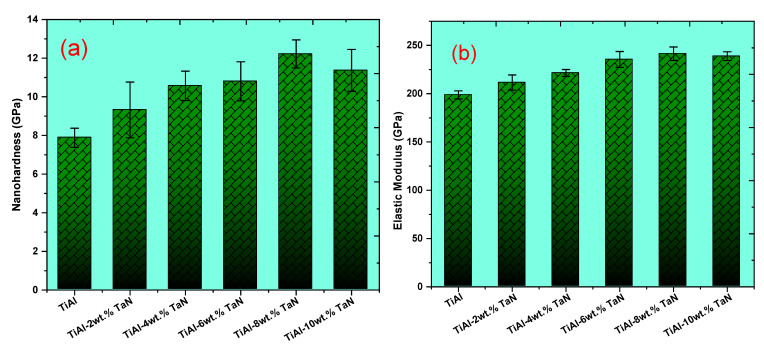
Nano-hardness (**a**) and elastic modulus (**b**) of the sintered TiAl and TiAl*_(100−x)_*-*_x_*TaN composites.

The nano-hardness and elastic modulus of the sintered TiAl and TiAl*_(100−x)_*-*_x_*TaN composites are presented in Figure 6a and Figure 6b, respectively. It can be observed that the sintered TiAl samples exhibit a nano-hardness value of 7.88 GPa, while the nano-hardness increases at the addition of 2, 4, 6, 8, and 10 wt.% TaN with a value of 9.32 GPa, 10.57 GPa, 10.8 GPa, 12.22 GPa, and 11.369 GPa, respectively. However, at 10 wt.% TaN addition, the nano-hardness value dropped, which is in agreement with the slight increment in the indentation depth in Figure 5b. The reduction in the nano-hardness value can be ascribed to two factors. On the one hand, it could be due to coalescence and segregation [52] or a poor metallurgical bond between the particles due to higher ceramic content [53,54,55,56]. On the other hand, ineffective sinterability is due to the prevailing sintering conditions. While the set sintering conditions may be feasible to attain dense and enhanced mechanical properties at lower TaN additions, higher sintering temperatures, for instance, may be required for enhanced sintering with higher TaN content. As obtained from this study, the highest nano-hardness value of 12.22 GPa was attained at the addition of 8 wt.% TaN, while the sintered TiAl exhibited the lowest hardness value of 7.88 GPa. The results obtained reflect the enhancement of the nano-hardness of the composites through the addition of TaN. The high hardness exhibited by the composites is directly related to their resistance to plastic deformation, as reflected by the load–depth curves (Figure 5a). Furthermore, the dislocation movement was impeded due to the addition of TaN, which facilitates the strengthening of the TiAl matrix.

Similarly, the elastic modulus of the sintered samples was presented in Figure 6b. The result obtained depicts a similar trend to the nano-hardness results (Figure 6a). As seen, the elastic modulus increases with increasing TaN addition. The highest elastic modulus was obtained at 8 wt. % addition of TaN with a value of 241.45 GPa, while the value dropped to 238.92 GPa at 10 wt.% TaN addition. The reduction in the elastic modulus of the composite with 8 wt.% TaN addition can be attributed to poor interfacial bonding between the particles [55]. In addition, the presence of increasing Al, Ti, and Ta fractions in the TiAl−10wt.% TaN composite, as presented in Figure 4, can be related to this poor bonding and consequent drop in mechanical properties. The enhanced elastic modulus displayed by the sintered material connotes a proportional increase in stiffness.

The enhanced nano-hardness and elastic modulus displayed by the sintered TiAl-based composites can be attributed to the formation of secondary phases, a reduction in crystallite size, and increased lattice strain via the addition of TaN, which helps to reinforce the mechanical properties.

#### 3.2.3. Mechanical and Anti-Wear Characteristics of the Sintered TiAl and TiAl*_(100−x)_*-*_x_*TaN Composites

**Figure 7:**
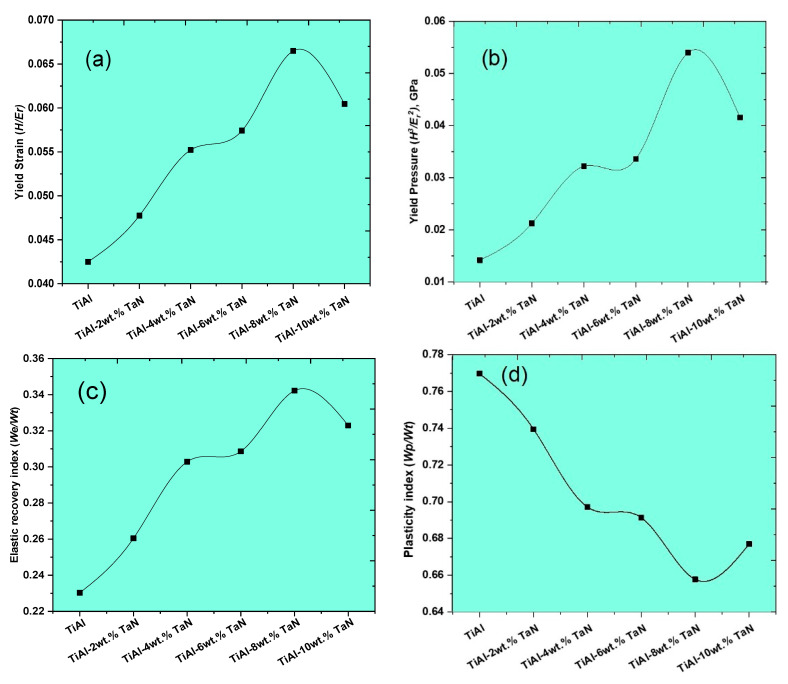

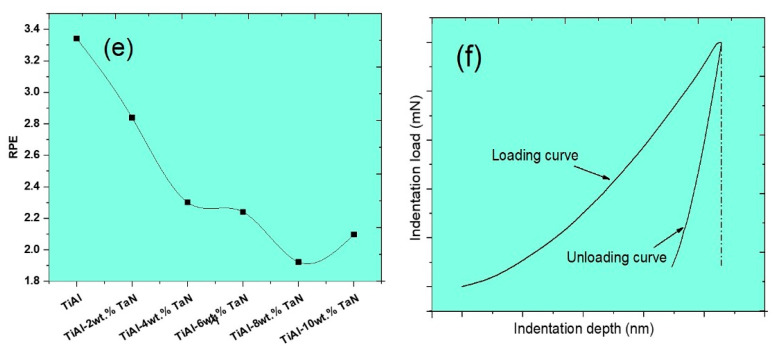
Yield strain (**a**), yield pressure (**b**), elastic recovery index (**c**), plasticity index (**d**), and RPE (**e**) of the sintered TiAl samples and composites at different TaN additions, and the schematic illustration of the load–depth curve and work carried out by indentation (**f**).

In addition to the indentation depth, nano-hardness, and elastic modulus properties of the sintered TiAl and composites, the elastic recovery index [WeWt], plasticity index [WpWt], and the ratio of plastic and elastic work (RPE), given by $RPE=\frac{\left(W_{t}-W_{e}\right)}{W_{e}}$ [57], which helps to determine the deformability of the sintered samples, were also evaluated. The anti-wear characteristics of the sintered materials were determined from the ratio of nano-hardness to the reduced elastic modulus, yield strain (HEr), elastic recovery index, plasticity index, and yield pressure (H3Er2) [44,49,58]. The elastic recovery index and the plasticity index indicate the energy utilized and the inherent plastic response in the material when subjected to indentation. Figure 7a,b present the yield strain and pressure of the sintered TiAl and Tial-based composites, which indicate the resistance of the sintered materials to elastic strain to failure and plastic deformation, respectively. The trend observed shows that the yield strain and pressure of the sintered materials increased as the TaN addition increased. A drop in the yield strain and pressure of the TiAl−10 wt.% TaN composite was observed, which corresponds to the reduction in the elastic and nano-hardness properties displayed in Figure 6. The highest yield strain and pressure of 0.0665 and 0.054 GPa, respectively, were attained by the TiAl−8wt.%TaN composite sample. Contrarily, the lowest yield strain and pressure were demonstrated by the TiAl samples with values at 0.0425 and 0.01422 GPa, respectively. The yield pressure (Figure 7b) shows that the sintered materials exhibited enhanced wear resistance as the TaN addition increased, with the maximum wear resistance attained at 8 wt.%TaN addition. The intrinsic plastic attributes of the sintered materials are demonstrated by the plasticity index. In contrast, the elastic recovery index is a representation of the energy released by the sintered material under load [49], and their corresponding deformability is evaluated using the RPE. As shown in Figure 7c,d, the sintered materials show that the elastic recovery index increases while the plasticity index decreases as the TaN addition increases, except at the addition of 8 wt.% TaN. The TiAl−8 wt.%TaN composite demonstrated the highest elastic index of 0.3423 and the lowest plasticity index of 0.6573, as shown in Figure 7c and Figure 7d, respectively. The RPE, as shown in Figure 7e, shows a similar trend to the plasticity index in Figure 7d. However, it shows that the deformability of the sintered materials reduces due to the increasing strengthening impact of TaN at increasing addition. The lowest RPE of 1.922 was demonstrated by the addition of 8 wt.% TaN, while TiAl exhibited the highest RPE of 3.342. The elasticity, plasticity, and deformability characteristics reflect the indentation resistance capabilities of sintered materials, as shown by the regions of loading and unloading curves covered in Figure 5a. This is schematically defined in Figure 7f, showing that the regions covered by the loading and vertical lines constitute the total deformation energy (*W_t_*) exhibited by the material, while the region between the loading and unloading curve denotes the plastic deformation energy (*W_p_*) exhibited by the material [31,59]. It can be seen in Figure 5a that the TiAl exhibited the highest deformation energy and the lowest was exhibited by the TiAl−8 wt.% TaN composite, which is corroborated by the trend observed in Figure 7d.

## 4. Conclusions

In this study, TiAl and TiAl*_(100−x)_*-*_x_*TaN composites were processed by the spark plasma sintering technique (SPS) at a temperature of 1150 °C, pressure of 50 MPa, 10 min dwell time, and at the heating rate of 100 °C/min. From the investigation, the following conclusions were drawn:

1. The TiAl comprises TiAl_2_, α-Ti, and TiAl phases with a duplex microstructure characterized by homogenous γ and lamellar colonies. With increasing additions of TaN, complex phases were formed, and the microstructure exhibited a typical pseudo-duplex structure;
2. The nano-hardness and elastic modulus increased as the TaN addition increased, as the highest values of 12.22 GPa and 241.45 GPa were obtained at the addition of 8 wt.%TaN, respectively. Subsequently, the highest yield strain, yield pressure, and elastic recovery index were demonstrated by the TiAl−8 wt.%TaN composite with values at 0.0665, 0.054 GPa, and 0.3423, respectively. In addition, the composite also demonstrated the lowest plastic index and RPE of 0.6573 and 1.922, respectively;
3. The TiAl−8wt.%TaN composites displayed the best mechanical properties. It is proposed that 8 wt.%TaN addition under the specified sintering condition is the optimal concentration for attaining the optimum mechanical properties of TiAl-based composites studied in this work.

## Figures and Tables

**Table 1:** Details of powders used for the fabrication of composites.

S/N	Powder	Purity (%)	Particle Size (µm)	Source
1	Ti-48Al-2Cr-2Nb	99.8	<36	TLS Technik, Bitterfeld, Germany
2	TaN	99	4–10	Cerac Inc., Milwaukee, WI, USA

**Table 2:** Details of the structural parameters of the sintered samples derived from the XRD pattern through the Williamson–Hall approach.

Sintered Material	Crystallite Size, D (nm)	Lattice Strain, *ɛ* (%)	$\mathrm{Dislocation}\;\mathrm{Density},\;\delta \;=\;\frac{1}{D^{2}}$
TiAl	24.7392	3.129	0.0016
TiAl−2wt.%TaN	21.4744	4.312	0.00217
TiAl−4wt.%TaN	21.186	6.444	0.0022
TiAl−6wt.%TaN	19.104	7.303	0.00274
TiAl−8wt.%TaN	15.1309	13.84	0.00437
TiAl−10wt.%TaN	13.510	16.271	0.00548

## Data Availability

The data utilized for this study are available upon request.
